# Impact of pretreatment plasma D-dimer levels and its perioperative change on prognosis in operable esophageal squamous cell carcinoma

**DOI:** 10.18632/oncotarget.18552

**Published:** 2017-06-16

**Authors:** Jianbo Li, Zhifan Zheng, Min Fang

**Affiliations:** ^1^ Department of Radiotherapy, Ningbo Mingzhou Hospital, Ningbo, China; ^2^ Department of Radiotherapy, Zhejiang Provincial People’s Hospital, Hangzhou, China

**Keywords:** esophageal squamous cell carcinoma, D-dimer, surgery, prognosis

## Abstract

The aim of this study was to investigate the relationship between plasma D-dimer levels and its perioperative change and clinicopathological parameters in patients with operable esophageal squamous cell carcinoma (ESCC). We also analyzed their prognostic significance in ESCC patients. The data of 294 ESCC patients between December 2007 and December 2012 in Mingzhou hospital, Ningbo, China were analyzed retrospectively. Plasma D-dimer levels were measured one week before surgery and on the thirtieth postoperative day. The association between plasma D-dimer levels and clinicopathological parameters was evaluated. Kaplan-Meier survival analysis and Cox proportional hazards models were used to estimate the effect of plasma D-dimer levels and its perioperative change on disease-free survival (DFS) and overall survival (OS). Plasma D-dimer levels were above 0.5 µg/mL in 148 patients (50.3%). Plasma D-dimer levels were significantly related with DFS (*P* < 0.001) and OS (*P* < 0.001) in univariate analysis. There was significant relationship between plasma D-dimer levels and DFS in patients with N_0_ (*P* < 0.001) or N_+_ (*P* = 0.003). Multivariate analysis revealed that plasma D-dimer levels (*P* < 0.001), sex (*P* = 0.012), and T stage (*P* = 0.033) were independent prognostic factors for DFS. Tumor length (*P* = 0.018), T stage (*P* = 0.008) and plasma D-dimer levels (*P* = 0.001) qualified as independent prognostic factors for OS. Our study suggests that pretreatment plasma D-dimer levels is a powerful independent prognostic factor for operable ESCC. Further studies are needed to prospectively validate this prognostic model and investigate the mechanisms underlying the correlation between elevated plasma D-dimer levels and poor prognosis in operable ESCC.

## INTRODUCTION

Esophageal cancer is the sixth leading cause of cancer-related mortality in both developing and developed countries [1]. Esophageal squamous cell carcinoma (ESCC) is the most prevalent type of esophageal cancer, particularly in East Asia and some parts of Europe [2]. Despite recently much more effort has been dedicated to improving treatment of patients suffering from ESCC, the prognosis remains quite poor, with a great number of patients experiencing disease progression in a short time [3]. Radical surgery is standard treatment that can provide opportunity for cure. However, the 5-year survival rates for surgically resectable ESCC are still unsatisfactory and range from 15% to 30% [4–6]. Adjuvant chemotherapy or radiotherapy could improve disease-free survival [7]. Furthermore, the benefit of platinum-based adjuvant chemotherapy for ESCC patients has not been established. Several studies have attempted to discover molecular biomarkers to predict the prognosis of ESCC [8–10]. However, to date, most of these markers had not been proven to be sufficiently effective [11].

D-dimer is a degradation product of fibrin in the blood during fibrinolysis, which could be used for diagnosis of thrombosis. Elevated plasma D-dimer levels were observed in acute venous thromboembolism, pregnancy, infectious diseases, as well as cancers [12, 13]. Furthermore, D-dimer has been implicated in tumor invasion, metastasis, and eventual worse outcome in various types of cancer [14, 15]. In ESCC, plasma D-dimer levels are useful for predicting lymph node metastasis [16]. In Tomimaru Y et al.’ report, plasma D-dimer levels correlated significantly with clinical and pathological responses to neoadjuvant chemotherapy [17]. However, the sample of these studies was relatively small. Furthermore, less studies have been performed to access the prognostic significance of postoperative plasma D-dimer levels after curative resection, which could reflect the patients’ status after tumor removal. Therefore, the aim of this study was to determine whether the plasma D-dimer levels before treatment and its perioperative change are predictors of mortality in patients with ESCC.

## RESULTS

### Patients

The baseline characteristics of 294 patients are presented in Table 1. 268 patients were male and 26 patients were female. The median age was 58 years (range: 38–70 years). 245 patients were former smoker. After surgery, 195 patients were detected to have T_3–4_ disease while 99 patients had T_1–2_ disease. There were 96 patients with N_0_ and 198 patients with mediastinum lymph node metastasis. The median tumor length was 4 cm (range: 0.5 cm–11.0 cm). Sixty patients (20.4%) received four to six cycles of cisplatin-based chemotherapy while sixty-two (21.1%) patients received adjuvant radiotherapy.

**Table 1:** Relationship between clinicopathological parameters, plasma D-dimer level and INR in patients with ESCC

Variables	Patients (*n*)	Plasma D-dimer level		*P*	Perioperative change of D-dimer level		*P*
		Normal (≤ 0.5 µg/ml)	High (> 0.5 µg/ml)		Decreased	Increased	
Gender							
Female	26	11 (42.3)	15 (57.7)	0.539	25 (96.2)	1 (3.8)	0.128
Male	268	135 (50.4)	133 (49.6)		229 (85.4)	39 (14.6)	
Age (years)							
< 65 Y	238	119 (50.0)	119 (50.0)	0.882	209 (87.8)	29 (12.2)	0.143
≥ 65 Y	56	27 (48.2)	29 (51.8)		45 (80.4)	11 (19.6)	
Smoking status							
Never	49	23 (46.9)	26 (53.1)	0.755	42 (85.7)	7 (14.3)	0.879
Ever	245	123 (50.2)	122 (50.2)		212 (86.5)	33 (13.5)	
Differentiation							
Well	32	24 (75.0)	8 (25.0)	0.025	30 (93.8)	2 (6.3)	0.168
Moderately	206	97 (47.1)	109 (52.9)		179 (86.9)	27 (13.1)	
Poorly	51	23 (45.1)	28 (54.9)		40 (77.4)	11 (21.6)	
Unknown	5	2 (40.0)	3 (60.0)		5 (100)	0 (0)	
Tumor location							
Upper	6	5 (83.3)	1 (16.7)	0.016	5 (83.3)	1 (16.7)	0.802
Middle	139	78 (56.1)	61 (43.9)		122 (87.8)	17 (12.2)	
Lower	149	63 (42.3)	86 (57.7)		127 (85.2)	22 (14.8)	
Tumor length							
≤ 5 cm	219	115 (52.5)	104 (47.5)	0.060	194 (87.8)	27 (12.2)	0.227
> 5 cm	73	29 (39.7)	44 (60.3)		60 (82.2)	13 (17.8)	
T stage							
T_1–2_	99	71 (71.7)	28 (28.3)	< 0.001	85 (85.9)	14 (14.1)	0.849
T_3–4_	195	75 (38.5)	120 (61.5)		169 (86.1)	26 (13.3)	
N stage							
N_0_	96	112 (56.6)	86 (43.4)	0.001	56 (58.3)	40 (41.7)	< 0.001
N_1–3_	198	34 (35.4)	62 (64.6)		188 (94.9)	10 (5.1)	

### Relationship between D-dimer and clinicopathological characteristics

The relationship between several clinicopathological characteristics, such as sex, age, tumor location, smoking status, tumor length, T stage and N stage are summarized in Table 1. Plasma D-dimer levels were above 0.5 µg/mL in 148 patients (50.3%). Tumor cell differentiation was related with plasma D-dimer levels (*P =* 0.025). Patients with low thoracic ESCC had higher plasma D-dimer levels, compared with upper or middle thoracic ESCC (*P =* 0.016). Besides, plasma D-dimer levels were also significant associated with T stage (*P <* 0.001) and N stage (*P =* 0.001). There were no correlation between plasma D-dimer levels and sex, age and smoking status (*P* > 0.05).

The clinicopathological features of two groups categorized by perioperative change of plasma D-dimer levels are summarized in Table 1. The patients’ distribution was 254 patients in D-dimer decreased group and 40 patients in D-dimer increased group. There were no significant differences among the perioperative change of plasma D-dimer levels with regard to sex, age, smoking status, tumor location, tumor length or T stage, whereas N stage showed significant difference (*P <* 0.001).

### Effect of plasma D-dimer levels on DFS and OS by survival analysis

During the follow-ups, 111 patients (37.8%) experienced distant organ metastasis, while 38 patients (12.9%) experienced local-regional recurrence. 97 patients (33.0%) died of disease progression finally. The 3-year DFS and OS rates were 48.9% and 59.7%, respectively. We performed univariate analysis for plasma D-dimer levels and other clinicopathological variables to find out the useful prognostic factors, which shown in Table 2. Sex (*P =* 0.031), T stage (*P <* 0.001), N stage (*P =* 0.012), plasma D-dimer levels (*P <* 0.001) and perioperative change of plasma D-dimer levels (*P =* 0.021) were significantly related with DFS in univariate analysis. Tumor length (*P =* 0.007), T stage (*P <* 0.001), N stage (*P =* 0.030), plasma D-dimer levels (*P <* 0.001) and perioperative change of plasma D-dimer levels (*P =* 0.026) were five significant prognostic factors related with OS. Patients with high plasma D-dimer levels had shorter DFS and OS than that with normal plasma D-dimer (3 year DFS rate: 36.8% Vs 62.9%, *P <* 0.001, Figure 1A; 3 year OS rate: 47.2% Vs 72.3%, *P <* 0.001, Figure 1B). Similarly, DFS and OS were significantly shorter in patients with increased plasma D-dimer level than in those with decreased plasma D-dimer level (3 year DFS rate: 36.5% Vs 51.0%, *P =* 0.021, Figure 2A; 3 year OS rate: 47.5% Vs 61.9%, *P =* 0.026, Figure 2B). In further analysis, high plasma D-dimer levels were significantly associated with shorter OS for T_1–2_ patients (*P =* 0.006) and for T_3–4_ patients (*P =* 0.005). Three year OS rate was 66.8% for high plasma D-dimer levels and 85.8% for normal plasma D-dimer levels in T_1–2_ patients, and 42.7% for high plasma D-dimer levels and 61.5% for normal plasma D-dimer levels in T_3–4_ patients. There was significant relationship between plasma D-dimer levels and DFS in patients with N_0_ (*P <* 0.001) or N_+_ (*P =* 0.003). Similarly, high plasma D-dimer levels were significantly associated with shorter OS for N_0_ patients (*P <* 0.001) and for N_+_ patients (*P =* 0.005). High plasma D-dimer levels were significantly associated with shorter OS for patients with adjuvant chemotherapy (*P =* 0.045) and for patients without chemotherapy (*P =* 0.019). There was significant difference between plasma D-dimer levels and OS in patients with postoperative radiotherapy (*P =* 0.032) or without postoperative radiotherapy (*P =* 0.029).

**Table 2:** Univariate analysis for clinicopathological parameters, plasma D-dimer levels, and perioperative change of D-dimer level associated with DFS and OS in patients with ESCC

Variables	3-year DFS rate	*P*	3-year OS rate	*P*
Sex				
Female	68.1	0.031	73.1	0.153
Male	47.0		57.9	
Age (years)				
< 65 Y	48.6	0.532	62.0	0.455
≥ 65 Y	50.3		51.8	
Smoking status				
Never	54.9	0.266	66.3	0.242
Ever	47.7		58.5	
Differentiation				
Well	56.5	0.699	65.5	0.392
Moderately	49.8		59.8	
Poorly	38.7		52.6	
Unknown	40.0		50.0	
Tumor location				
Upper + Middle	55.0	0.095	66.8	0.102
Lower	42.6		52.4	
Tumor length				
≤ 5 cm	50.7	0.126	62.3	0.007
> 5 cm	41.4		51.1	
T stage				
T_1–2_	67.2	< 0.001	78.0	< 0.001
T_3–4_	39.6		49.3	
N stage				
N_0_	55.0	0.012	65.2	0.030
N_1–3_	37.8		49.3	
Adjuvant chemotherapy				
Yes	36.9	0.175	54.9	0.627
No	52.6		61.1	
Adjuvant radiotherapy				
Yes	41.2	0.286	54.1	0.090
No	51.2		61.3	
Plasma D-dimer level				
Normal (≤ 0.5 µg/ml)	62.9	< 0.001	73.9	< 0.001
High (> 0.5 µg/ml)	36.8		47.2	
Perioperative change of D-dimer level				
Decreased	51.0	0.021	61.9	0.026
Increased	36.5		47.5	

Abbreviations: DFS, Disease-free survival; OS, Overall survival; ESCC, Esophageal squamous cell carcinoma.

**Figure 1A:**
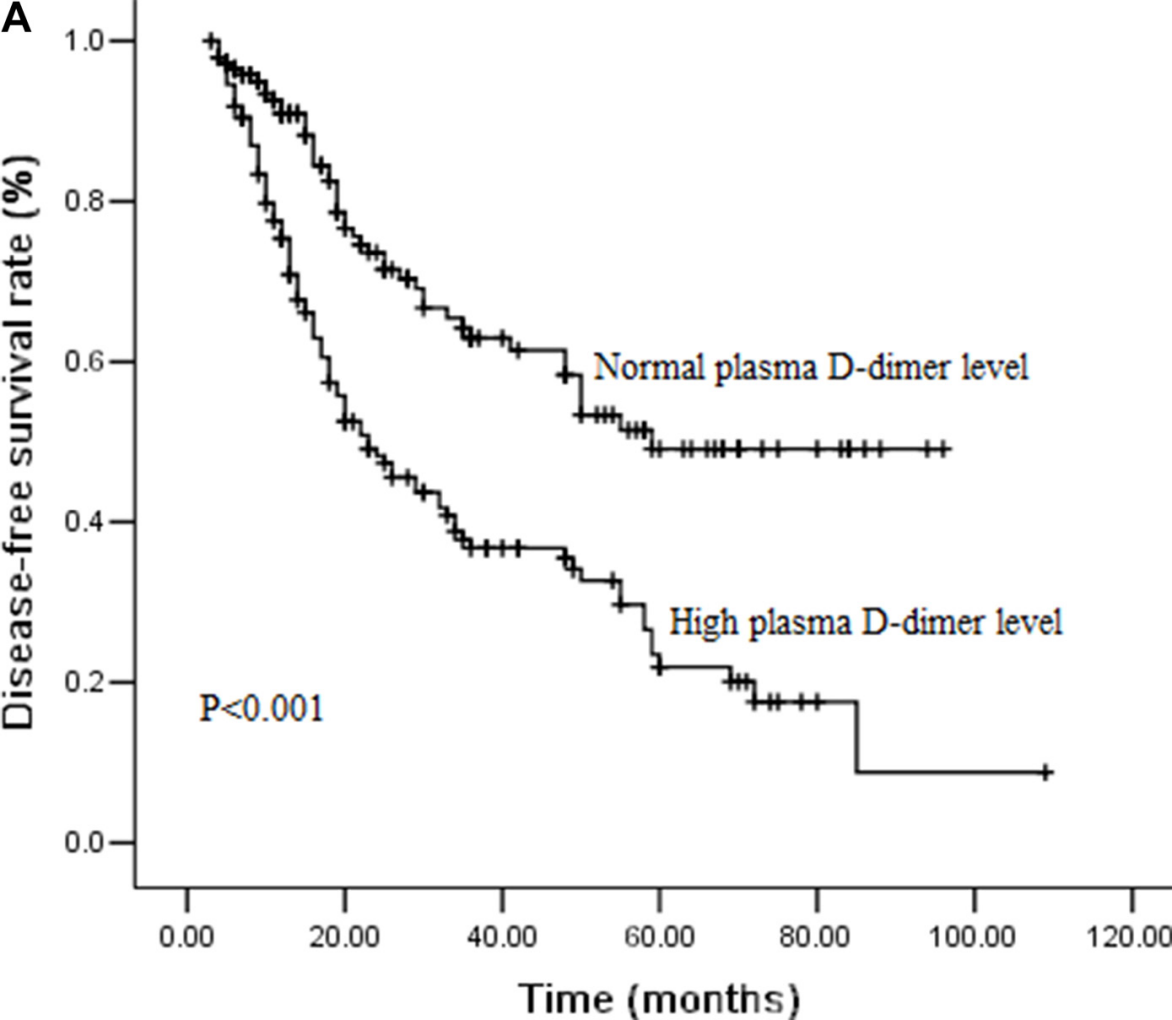
Cumulative survival curves for disease-free survival (DFS) time according to pretreatment plasma D-dimer levels

**Figure 1B:**
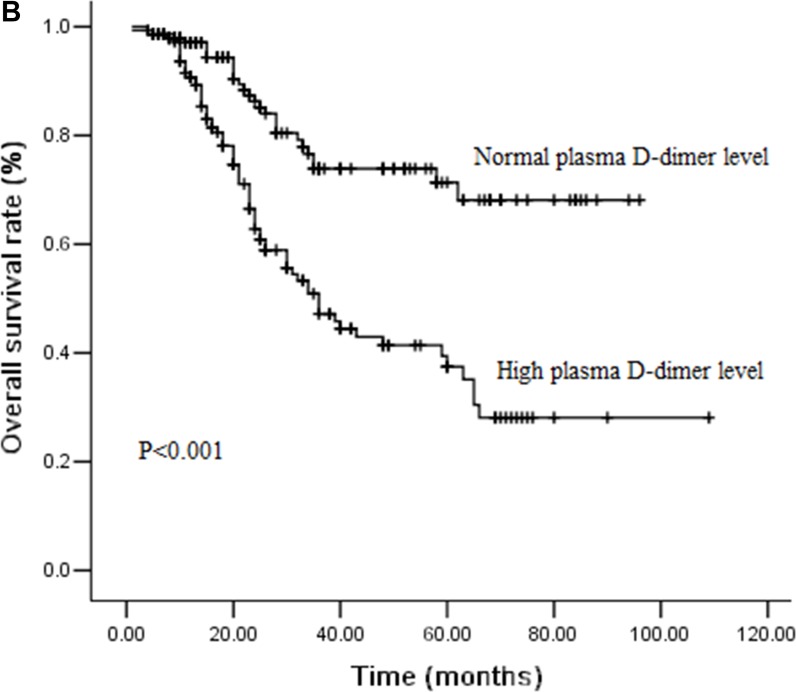
Cumulative survival curves for overall survival (OS) time according to pretreatment plasma D-dimer levels

**Figure 2A:**
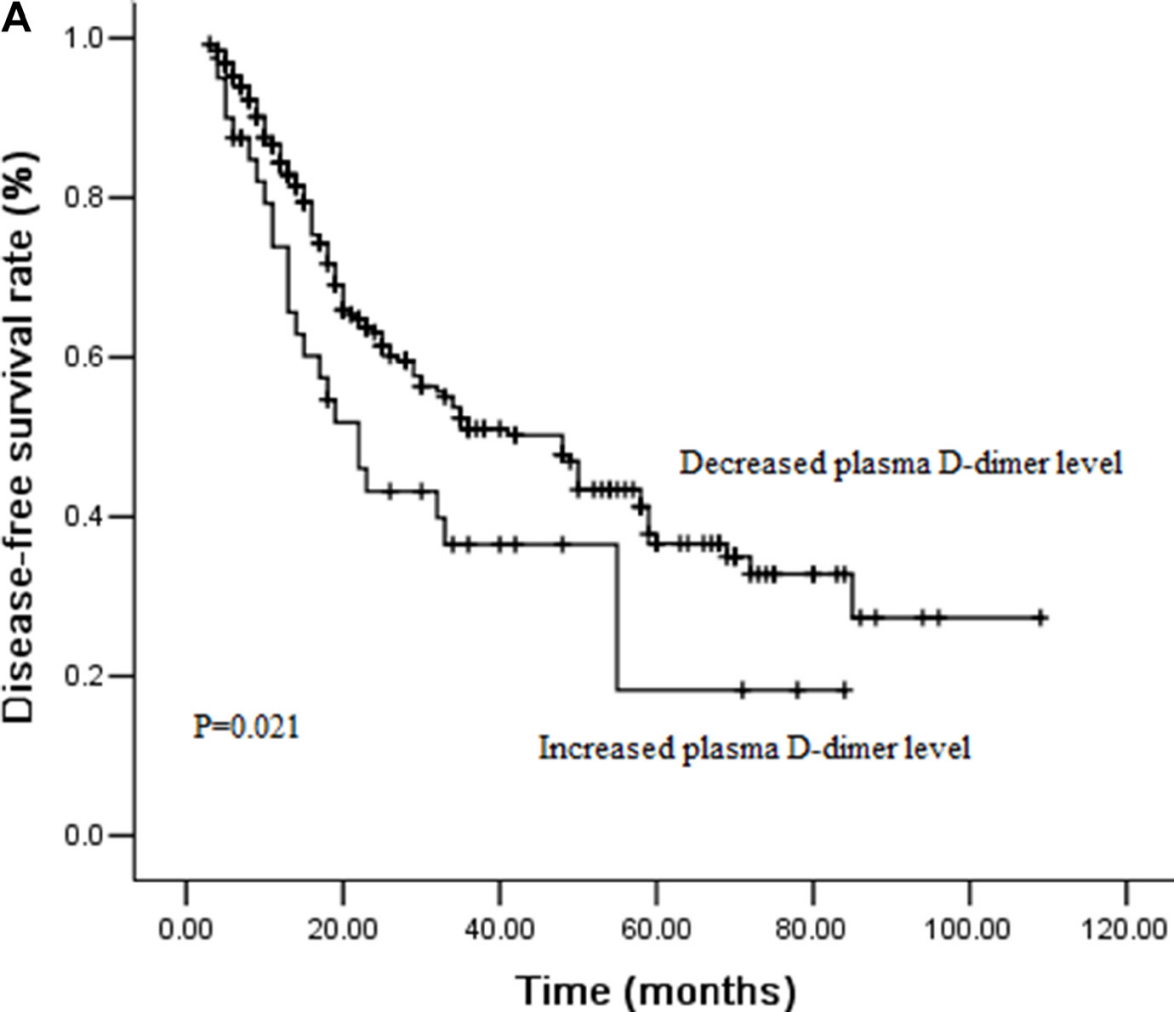
Cumulative survival curves for disease-free survival (DFS) according to perioperative change of plasma D-dimer levels

**Figure 2B:**
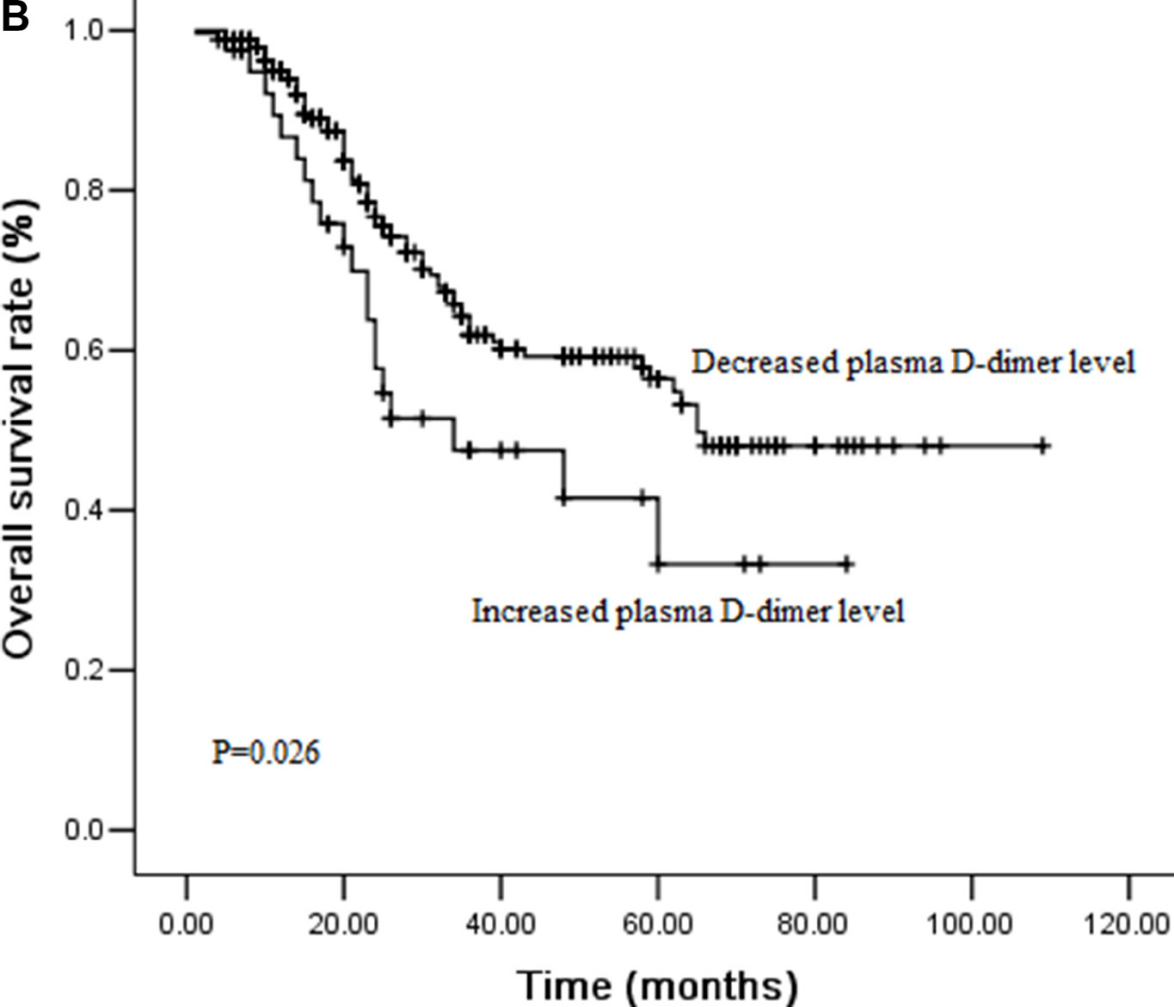
Cumulative survival curves for overall survival (OS) time according to perioperative change of plasma D-dimer levels

Then, we performed multivariate analysis on the factors that were statistically significant in the univariate analysis. The results are shown in Table 3. The Cox proportional hazards regression indicated that plasma D-dimer levels (*P <* 0.001), sex (*P =* 0.012), and T stage (*P =* 0.033) were independent prognostic factors for DFS. Tumor length (*P =* 0.018), T stage (*P =* 0.008) and plasma D-dimer levels (*P =* 0.001) qualified as independent prognostic factors for OS. Patients with high plasma D-dimer levels had an elevated risk of disease progression and death compared to those with normal plasma D-dimer levels. The hazard ratio was 2.11 (95% confidence interval [CI] 1.46–3.05) for disease progression and 2.25 (95% CI 1.42–3.56) for death.

**Table 3:** Results of multivariate analyses of clinicopathological factors affecting DFS and OS in patients with ESCC

Variables	DFS			OS		
	RR	95% CI	*P*	RR	95% CI	*P*
Sex (male Vs female)	2.47	1.19–5.13	0.016	-	-	-
Tumor length (> 5 cm Vs ≤ 5 cm)	-	-	-	1.66	1.07–2.57	0.023
T stage (T_3–4_ Vs T_1–2_)	1.59	1.04–2.42	0.033	2.20	1.25–3.85	0.006
N stage (N_1–3_Vs N_0_)	1.13	0.73–1.75	0.571	1.03	0.61–1.74	0.926
Plasma D-dimer level (High Vs normal)	2.09	1.43–3.05	< 0.001	2.23	1.39–3.57	0.001
Perioperative change of D-dimer level (Increased Vs Decreased)	1.07	0.61–1.86	0.813	1.28	0.67–2.43	0.451

Abbreviations: DFS, Disease-free survival; OS, Overall survival; ESCC, Esophageal squamous cell carcinoma; RR, relative risk; CI, confidence interval.

## DISCUSSION

Hypercoagulability in patients with malignant tumors of gastrointestinal tract system is common and well known. In a large scale of 1042 gastric patients, plasma D-dimer levels were significantly elevated in metastasis GC patients, especially in patients with hematogenous visceral metastasis, and plasma D-dimer levels correlated with vascular cancer emboli in resected tissue samples [18]. In this present study, we demonstrated that plasma D-dimer levels were significant related with T stage (*P <* 0.001) and N stage (*P =* 0.001). Patients with low thoracic ESCC had higher plasma D-dimer levels, compared with upper or middle thoracic ESCC (*P =* 0.016). We also showed that high plasma D-dimer levels were significantly associated with decreased DFS and OS probability. Patients with elevated plasma D-dimer levels had 2.11 times the risk for disease progression and 2.25 times the risk for death compared with those with normal plasma D-dimer levels.

Diao et al. [19] examined plasma D-dimer levels between 66 patients with esophageal cancer and 12 patients with benign disease before and after operation. Plasma D-dimer levels were increased significantly higher in cancer group. OS time was significantly short in cancer patients whose plasma D-dimer levels on the 3rd and 9th post-operative day above the median number. In another report [16], plasma D-dimer levels were associated with the number of lymph node metastasis (*P <* 0.001). Compared with other serum tumor markers, such as carcinoembryonic antigen (CEA) and squamous cell carcinoma–related antigen (SCC-Ag), D-dimer is the best marker to predict N stage before operation. However, the sample of this study was relatively small and the author did not clarify the correlation between hematogenous metastasis and D-dimer. Tomimaru Y et al. [17] investigated the association between plasma D-dimer levels and chemotherapy response. Plasma D-dimer levels were significantly lower in patients response to chemotherapy. Although these results somewhat overlapped with our study, the strength of the current study was direct comparison of plasma D-dimer levels ESCC under the same treatment. We also performed sub-group analysis to investigate the role of plasma D-dimer levels in ESCC. Furthermore, patients were categorized into two groups according to their plasma D-dimer levels’ variation before and after operation: Increased = plasma D-dimer levels increasing in the perioperative period; Decreased = plasma D-dimer levels decreasing in the perioperative period. Effect of perioperative change of plasma D-dimer levels on DFS and OS was evaluated by survival analysis.

Our study also had several limitations. First, plasma D-dimer levels and platelet counts were checked only once in each patient and were not examined during the treatment and the follow-up. The relationship between changes in plasma D-dimer levels and tumor progression need to be investigated. Second, our study included a comparative homogeneous population with the majority of male and smoker patients, which might cause a bias. Also, this is a retrospective study based on patients of one institution and could not completely avoid selection bias. Furthermore, distant organ metastasis rate during follow-up was not the focus in this study, they should be reported. But unfortunately, we do not have detailed information of metastasis due to the retrospective nature of this study.

## MATERIALS AND METHODS

### Patients

In this study, the data of 294 ESCC patients between December 2007 and December 2012 in Mingzhou hospital, Ningbo, China were analyzed retrospectively. All patients were newly confirmed to have ESCC and had not received treatment previously. Patients with other malignancies were excluded from this study. Pretreatment plasma D-dimer levels and serum CRP levels obtained within 1 week before surgery. Patients with a history of venous thrombosis or anticoagulation therapy, hypertension, cardiovascular and cerebrovascular disease and diabetes were excluded from the current study. The following detail clinical information was retrospectively collected and analyzed for each case: sex, age at treatment, smoking status, tumor location, clinical TNM stage, treatment response, disease-free survival (DFS) and overall survival (OS) after treatment. TNM classification was evaluated according to the 7th edition of the Union for International Cancer Control staging. All procedures performed in studies involving human participants were in accordance with the ethical standards of the institutional and/or national research committee. Informed consent was obtained from all individual participants included in the study.

### D-dimer measurement

As a part of clinical routine examinations, plasma D-dimer levels were measured 1 week before surgery and on the thirtieth postoperative day. Each patient provided two 5 mL blood samples before breakfast. Plasma D-dimer levels were measured by a latex-enhanced immunoturbidimetric assay using a Sysmex CA 7000 (Sysmex Corp, Kobe, Japan) analyzer according to manufacturer’s instruction in Mingzhou hospital. Plasma D-dimer levels of 0.5 μg/mL was used as cutoff for normal versus high D-dimer values, according to the manufacture’s recommendation. Furthermore, patients were categorized into two groups according to their plasma D-dimer levels’ variation before and after operation: Increased = plasma D-dimer levels increasing in the perioperative period; Decreased = plasma D-dimer levels decreasing in the perioperative period.

### Treatment

All patients underwent total or subtotal transthoracic esophagectomy and regional lymphadenectomy with curative intent. Sixty (20.4%) were treated with adjuvant platinum-based chemotherapy and sixty-two (21.1%) patients received post-operative radiotherapy. All patients received standardized follow-up at a 3-month interval for the first 2 years after operation, a 6-month interval in the third year and yearly thereafter. The median follow-up time was 26 months.

### Statistical analysis

Disease-free survival (DFS) was defined as the time from surgery to any recurrence, including locoregional recurrence and distant organ metastasis. Overall survival (OS) was calculated as the time from the date of surgery to death or censoring. The chi-square test was performed to evaluate the association between the clinicopathological variables and plasma D-dimer levels and INR. Survival curves were estimated by the univariate Kaplan-Meier method. The log-rank test was applied to check the significant difference in the curves among groups. Furthermore, we used the Cox proportional hazards model for multivariate analysis. All statistical calculations were performed with SPSS 23.0 software for Windows (Chicago, IL, USA). Two-sided *P*-values of < 0.05 were considered to be statistically significant.

## CONCLUSIONS

In conclusion, we have provided the clinical evidence that plasma D-dimer levels were related with clinicopathological factors in ESCC. We demonstrated that pretreatment plasma D-dimer levels could be served as new independent prognostic biomarkers for DFS and OS in operable ESCC, although its prognostic significance still requires confirmation with larger patient populations.
